# Recovery from Apparent Death by Cold; Remarkable Effect of Birchtar

**Published:** 1845-09

**Authors:** 


*Recovery from apparent death by cold; remarkable effect of birch-tar.*—The body of a man, ætat. 45, was discovered, perfectly stiff and cold, but not entirely frozen; his mouth could not be opened by any means; no sign of respiration or arterial action could be detected. After the body had been softened by frictions with snow, rags dipped in cold water, and woollen cloths, the lungs were inflated, and clysters of tobacco were administered without effect; as a last resort, the author placed the body on a straw mattrass, with the head raised, and ordered the sexual organs, particularly the scrotum and perinæum, to be well rubbed with birch-oil, when very soon a sound like bleating was heard; and after the frictions had been extended to the abdomen and upper parts of the vertebral column, furthe signs of life appeared. He was then laid in a warm bed, where he soon recovered his senses, and fell into a sound, healthy sleep. The birch-oil is very much used in Russia as a domestic remedy, both to sober drunken people and to cause them to dislike spirituous liquors. In both cases the sexual organs are rubbed with it, and a violent headache is said to arise, which lasts for a day or two. It is also taken internally, as a popular remedy for dropsy and rheumatism.—*Ibid, from Dr. Kostroff in Medic. Zeitung Russlands.*

